# Relationship between Gut Microbiota and Allergies in Children: A Literature Review

**DOI:** 10.3390/nu15112529

**Published:** 2023-05-29

**Authors:** Alexandru Cosmin Pantazi, Cristina Maria Mihai, Adriana Luminita Balasa, Tatiana Chisnoiu, Ancuta Lupu, Corina Elena Frecus, Larisia Mihai, Adina Ungureanu, Mustafa Ali Kassim Kassim, Antonio Andrusca, Maria Nicolae, Viviana Cuzic, Vasile Valeriu Lupu, Simona Claudia Cambrea

**Affiliations:** 1 Pediatrics, Faculty of General Medicine, “Ovidius” University, 900470 Constanta, Romaniacristina2603@yahoo.com (C.M.M.);; 2 Pediatrics, County Clinical Emergency Hospital of Constanta, 900591 Constanta, Romania; 3 Pediatrics, “Grigore T. Popa” University of Medicine and Pharmacy, 700115 Iasi, Romania; valeriulupu@yahoo.com; 4 Faculty of General Medicine, “Ovidius” University, 900470 Constanta, Romania; 5 Infectious Diseases, Faculty of General Medicine, “Ovidius” University, 900470 Constanta, Romania

**Keywords:** allergy, asthma, atopic dermatitis, children, food allergy, microbiota

## Abstract

The intestinal microbiota is a diverse and complex microecosystem that lives and thrives within the human body. The microbiota stabilizes by the age of three. This microecosystem plays a crucial role in human health, particularly in the early years of life. Dysbiosis has been linked to the development of various allergic diseases with potential long-term implications. Next-generation sequencing methods have established that allergic diseases are associated with dysbiosis. These methods can help to improve the knowledge of the relationship between dysbiosis and allergic diseases. The aim of this review paper is to synthesize the current understanding on the development of the intestinal microbiota in children, the long-term impact on health, and the relationship between dysbiosis and allergic diseases. Furthermore, we examine the connection between the microbiome and specific allergies such as atopic dermatitis, asthma, and food allergies, and which mechanisms could determine the induction of these diseases. Furthermore, we will review how factors such as mode of delivery, antibiotic use, breastfeeding, and the environment influence the development of the intestinal flora, as well as review various interventions for the prevention and treatment of gut microbiota-related allergies.

## 1. Introduction

The gut microbiota represents the population of microorganisms that inhabit the human gut. Over the last decade, several studies have been conducted to determine the relationship between the microbiota and allergies in children. The findings suggest that the gut microbiota has a significant role in the promotion of these allergies. For instance, Penders et al. [1] conducted a comprehensive assessment of 18 studies investigating the connection between the microbiota of the gut and allergic diseases. These studies, published between 1999 and 2006, were mainly observational and compared the characteristics of the microbiota in allergic diseases. They analyzed the gut microbiota profiles of subjects with various allergic conditions, including atopic dermatitis, wheezing, food allergy, allergic rhinitis, and asthma. The methods used to evaluate the microbiota composition varied from traditional bacterial cultures to advanced molecular biology techniques. Most studies found a correlation between the composition of the microbiota and the presence of allergic clinical manifestations. However, it was difficult to differentiate between protective microorganisms and those linked to an increased risk of allergic diseases. Variations in study types and laboratory techniques used to evaluate the microbiota composition were attributed to differences in results [1]. 

Similarly, Melli et al. [2] conducted a study which analyzed research published between 2007 and 2013 and included 21 studies that examined the composition of the gut microbiota in allergic conditions. It was observed that compared to nonallergic children, those with allergies presented a lower level of biodiversity in their colonic microbiota, characterized by an overabundance of *Firmicutes* and a higher count of *Bacteroidaceae*. Another study conducted by Azad et al. in 2013 found that infants with a lower diversity of gut microbiota are at a higher risk of developing allergies later in life [3]. Other studies have stated that alterations in the composition of the microbiota have been linked to the onset of various diseases [4,5]. Additionally, a recent study published in 2022 found that breastfeeding is associated with a decreased risk of food allergies in children, due to beneficial effects of breast milk on the gut microbiome [6].

## 2. Development of the Gut Microbiota in Children

A study conducted by Odamaki et al. [7] in 2016 revealed that the gut microbiota undergoes age-related changes. Stool samples were analyzed from 367 healthy Japanese individuals ranging from 0 to 104 years of age using 16S rRNA sequencing. It was observed that the microbiota composition remained stable during adulthood, with *Firmicutes*, including *Lactobacillus* and *Clostridium*, being the most prevalent phylum in the intestinal microbiota among adult subjects. On the other hand, *Actinobacteria*, including *Bifidobacterium*, were more abundant in samples obtained from one-year-old participants, with their relative abundance decreasing after the weaning period. The intestinal microbiota developed to resemble an adult-like gut microbiota by the age of three. 

Many studies have reported that the establishment of the human gut microbiota begins in fetal life through various sources; one of them is the detection of bacterial DNA in the placenta [8]. It is worth noting that there is ongoing debate among the scientific community regarding its presence in this organ (with some studies reporting the presence of bacterial DNA and/or live bacteria in the placenta, while others have failed to find conclusive evidence of a placental microbiome) [9,10]. These conflicting results have led to suggestions that any bacterial presence observed in the placenta could potentially be attributed to contamination during the collection or processing of samples. Furthermore, bacterial DNA has been found in the amniotic fluid [11] and meconium of children born by cesarean section, providing strong evidence for the colonization of the gut microbiota during early life [12]. After delivery of the fetus, it will come in contact with many different flora that will increase the population of the microecosystem. This has been observed in a study evaluating the bacterial quantity in the infant gut of subjects with vaginal delivery, who acquired abundant bacteria present in the vaginal and perianal area, which accelerates colonization of the intestinal microbiota as established through examining the gut microbiota of infants. A level of 107 bacteria per gram of stool on day 1 of life was reported, which increased to 109 per gram on day 3, 1010 per gram on day 7, and 1011 per gram by 6 months, almost reaching the level found in adults [13].

## 3. Factors Influencing Microbiome Development

The role of host genotype in shaping the composition of gut bacteria has only been acknowledged in recent times. To investigate the genetic factors involved, the traditional approach used is to compare data between monozygotic twins (MZ) and dizygotic (DZ) twins [14]. An extensive study conducted on twins (*n* = 416) reported that monozygotic twins have a more similar gut microbiota composition than dizygotic twins, highlighting the influence of genetic factors on the intestinal microbiome. Additionally, this study identified several heritable bacterial species, with the most heritable belonging to the family of twins [15]. Two years after the initial study, the same study group tripled the sample size with 1126 twin pairs. This larger cohort study validated previously discovered heritable bacteria and revealed novel associations between host genes and bacterial strains [16]. 

Another significant factor influencing microbiome development is the mode of delivery [17]. Infants born by vaginal delivery acquire bacterial species from the vaginal and perianal area such as *Lactobacillus*, *Prevotella*, or *Sneathia* spp. [18], while infants delivered via caesarean section have reduced exposure to these bacteria [19], resulting in a different composition of their microbiome [20]. 

Breastfeeding is also essential to shape the microbiome of infants. Breast milk contains various prebiotics, such as human milk oligosaccharides, which selectively promote the growth of beneficial bacteria such as *Bifidobacterium* and *Lactobacillus* [21]. However, infants who are fed with milk formulas have microbiomes such as *Roseburia*, *Clostridium*, and *Anaerostipes* [19]. Dietary factors also have another effect on the microbiome, such as high-fiber diet, which promotes the development of fiber-degrading bacteria, leading to a more diverse and stable microbiome [22]. Antibiotic use during infancy and early childhood has also been linked to alterations in the microbiome composition, potentially leading to dysbiosis [23]. Gestational age represents another determining factor; the preterm intestine is colonized mainly by *Enterobacter*, *Staphyloccoccus*, and *Enterococcus*, while in a full-term infant the colonization is mainly by *Bacteroides*, *Bifidobacterium*, *Parabacteroides*, and *Escherichia* [24]. Furthermore, environmental factors such as exposure to pets, urbanization, and sanitation can affect the microbiome, with increased exposure to microbial diversity generally associated with a more diverse composition [25]. 

The combination of these factors can disrupt the balance of the intestinal microbiota and cause dysbiosis, in some cases causing cardiac pathology such as heart failure and correlated with the severity of the disease [26,27]. Heart failure is associated with significant changes in the gut microbiome [28]. These changes include a reduction in core intestinal microbiota, decreased bacterial diversity, increased levels of potentially harmful bacteria, and a decline in the production of short-chain fatty acids [28]. Furthermore, some individuals with heart failure may present an increased intestinal permeability, allowing bacterial products to enter the bloodstream and contribute to disease progression. The microbiota plays an important role in immunity. Therefore, dysbiosis can create an environment that is more favorable for the growth and spread of harmful microorganisms such as *Shigella* spp. A retrospective study over a 10-year period, conducted on 376 patients with *Shigella* [29], revealed that children under five years old were more susceptible to *Shigella* spp. The study also found that atmospheric temperature, humidity, and rainfall were significant environmental factors influencing the incidence of *Shigella* spp. Similarly, in a retrospective study spanning a decade, from 1 January 2009 to 31 December 2018, researchers examined 377 patients diagnosed with *Salmonella* spp. disease [30]. The study findings indicate a significant correlation between the occurrence of *Salmonella* spp. cases and elevated humidity and atmospheric temperature levels. These environmental factors could have initiated dysbiosis which led to the child intestine vulnerability to *Salmonella* species. However, there is a limited amount of published research to support the hypothesis.

## 4. Microbiome, Obesity, and Body Health

The microbiome is involved in various aspects of body health in children. For example, analyses have illustrated that the microbiome is taking part in the development and maturation of the immune system in children [31,32,33]. The gut microbiome has been shown to play a critical role in immune system development, as it is involved in the production of immunoglobulins and other immune system components [34]. A study published by Blanton et al. [35], has shown that the configuration of the intestinal microbiota during childhood can have a significant impact on body growth and development, and that underweight children presented a less-diverse gut microbiota compared to healthy children. The researchers suggested that the less-diverse microbiota could lead to poor nutrient absorption, resulting in stunted growth. The gut microbiota in individuals with obesity has been found to have a heightened ability to ferment polysaccharides from the diet, which are typically indigestible by the host. This results in increased absorption of monosaccharides and short-chain fatty acids (SCFA), promoting the liver conversion of complex lipids and subsequent deposition of adipocytes [36]. 

Research has revealed a correlation between dysbiosis, elevated levels of SCFA, obesity, and metabolic alterations. However, the precise connection between SCFAs and obesity remains uncertain [37,38]. SCFAs, which are produced by the intestinal microbiota, play a fundamental role in regulating intestinal permeability, bile acid metabolism, inflammation, and immune functions. In individuals with obesity, it is suggested that an increased production of colonic SCFAs allows for greater microbial energy harvest. However, certain SCFAs can also activate specific peptide hormones, stimulating feelings of satiety and promoting glucose disposal in peripheral tissues [38]. A study conducted by Ley et al. [39] reported that the gut microbiota of obese individuals was distinguished by a greater proportion of *Firmicutes* and a lesser proportion of *Bacteroidetes* compared to underweight subjects. Another study by Liu et al. [40] determined the association between dysbiosis and obesity. A more recent study investigated the effects of a high-fat diet on the gut microbiota of human subjects. The authors found that the high-fat diet resulted in alterations in the gut microbiota that were associated with increased obesity [41]. In conclusion, the intestinal microbiota maintains a vital part in the development of obesity. Dysbiosis and altered constituents of the intestinal microbiota in individuals with obesity promote the fermentation of indigestible dietary polysaccharides and the absorption of SCFAs, ultimately leading to an increased deposition of adipocytes. More research is needed to completely understand the complex connection between SCFAs, the gut microbiota, and obesity.

## 5. The Relationship between Dysbiosis and Allergic Diseases

Dysbiosis, defined as an imbalance or maladaptation in the microbiota, is increasingly recognized as a significant factor in the development of allergies in children [42]. The normal population of the intestinal microbiota aids in crucial physiological processes such as digestion [43], metabolism [44], and immune system regulation [45]. The complex interplay between gut microbiota dysbiosis and the development of allergic diseases has recently emerged as a topic of significant scientific interest [46,47,48]. In 2017, the first discovery was made by biologist Erik Wambre and immunologist William Kwok, who found that a specific type of cell, known as T helper type 2 cell, which produces Interleukin-4 (IL-4), Interleukin-5 (IL-5), Interleukin-9 (IL-9) and Interleukin-13 (IL-13), plays a critical role in triggering allergic reactions [49]. This was further demonstrated in February 2018 by multiple studies which determined a direct connection between T helper type 2 cells and allergen sensitization in allergic rhinitis [50]. Regardless of research spanning more than two decades on the use of immune molecules to prevent allergic diseases, no effective strategies have been established yet [51]. The composition of the gut microbiota is understood to be intrinsically linked to the maturation and regulation of the host’s immune system, thus any perturbations in this delicate balance, such as those caused by dysbiosis, can potentially result in abnormal immune responses and, subsequently, allergic diseases [33,52]. The “hygiene hypothesis” puts forth that a diminished exposure to commensal and pathogenic microorganisms during early childhood may lead to a lack of adequate immune system stimulation and maturation [53]. In this context, dysbiosis may serve as a critical factor in the increasing prevalence of allergies. Furthermore, certain bacterial species, including *Bifidobacteria* and *Lactobacilli*, play an essential role in sustaining immune homeostasis [54,55]. Their contribution to the stimulation of regulatory T-cells that can mitigate allergic responses, along with the promotion of anti-inflammatory cytokines such as IL-10, is significant [56]. Dysbiosis often results in a reduction of these crucial species, which can disrupt immune equilibrium and predispose individuals to allergic reactions. Additionally, a primary factor contributing to dysbiosis probably will be reduction in gut microbiota diversity, leading to decreased resistance to pathogenic microorganisms and immune system weakening [57]. This can potentially result in allergic disease development. Moreover, dysbiosis can lead to an increase in intestinal barrier dysregulation, allowing allergens to enter the bloodstream and trigger an immune response thought to occur due to the release of inflammatory mediators and cytokines (e.g., IFN-γ, TNF-α) that lead to degradation of the intestinal barrier [58,59]. Lastly, an interesting new hypothesis suggested that dysbiosis resulting from various factors including cesarean delivery and antibiotic use leads to a decrease in butyric-acid-producing bacteria (BAPB), which leads to a decrease in intestinal butyric acid concentrations [60]. The decrease in butyric acid concentration can suppress the differentiation of T-cells into regulatory T-cells (Tregs). The reduced number of Tregs impairs the immune system’s ability to control excessive immune responses, thereby contributing to the onset of allergic diseases [60]. Some previous studies support this hypothesis, with one study showing that children with high levels of butyric acid in their stool samples at 18 months of age tend to have fewer sensitized allergens [61]. Following the hypothesis, prebiotics and probiotics can increase the levels of BAPB, and postbiotics that are rich in butyric acid could be a promising preventive or therapeutic approach to allergic diseases. Postbiotics are bioactive compounds released through the metabolic activity of microorganisms, and they can have beneficial effects on the host [62].

## 6. Interventions for the Prevention and Treatment of Gut Microbiota-Related Allergies

Consumption of live microbes known as probiotics in adequate amounts is known to provide health benefits to the host [63]. The probiotics therapeutic mechanism primarily hinges on the restoration of the microbial equilibrium within the gastrointestinal tract [64]. They achieve this by competitive exclusion of pathogenic bacteria, improvement of the gut barrier function, and modulation of the host immune response [65,66,67]. Competitive exclusion involves outcompeting harmful bacteria for nutrients and adhesion sites on the gut epithelial surfaces, thus limiting their proliferation [68]. Improving the gut barrier function is essential in mitigating translocation of pathogens and their toxins across the intestinal wall, thereby reducing systemic inflammation [66]. Finally, probiotics can modulate the immune response by enhancing the production of anti-inflammatory cytokines (e.g., IL-10, TGF-β) and reducing the secretion of proinflammatory ones (e.g., TNF-α, IL-6), thereby promoting a balanced gut immune response [69]. Probiotics have been found to be effective in preventing and treating various disorders related to the gut microbiota, such as antibiotic-associated diarrhea [70], inflammatory bowel disease [71], celiac disease [72] and other allergies [73]. A study conducted by Fassio et al. [74] about allergic rhinitis, which was a review of ten studies, reported that five studies showed a significant decrease in symptom scores and an improvement in quality of life of patients with allergic rhinitis, which indicate a positive effect of probiotics. *Bifidobacterium* and *Lactobacillus* are among the most important microorganisms in the early life of children and they appeared to be effective in treating allergic rhinitis in the previous article. More research is required to confirm these findings. 

Prebiotics, which are non-digestible components that exist within food such as fibers and carbohydrates, selectively promote the growth and the activity of the microecosystem that exist in the human gut. Prebiotics function as substrates, selectively stimulating the growth and activity of health-promoting bacteria such as *Bifidobacterium* and *Lactobacillus* species [75]. When consumed, prebiotics bypass digestion in the upper gastrointestinal tract, reaching the colon intact, where they undergo fermentation by resident microbiota, producing short-chain fatty acids (SCFAs), including butyrate, propionate, and acetate [76]. These SCFAs have numerous health benefits, including improving the gut barrier function, enhancing immune response, and regulating host metabolism, thereby aiding in the restoration of a balanced microbiota composition [77]. These components have been studied as a potential intervention for conditions related to gut-microbiota-related pathology [78]. They have also been shown to have a positive effect in reducing the risk of developing allergies; one example of this is a study which was conducted on infants which showed that prebiotic supplementation reduced the risk of atopic dermatitis by 50% compared to placebo treatment [79]. 

Fecal microbiota transplantation is a method which can normalize the gut microbiota of the affected individuals through replacement of that microbiota with another microecosystem from a healthy individual [80], and it has been proving effective in treating many conditions such as recurrent *Clostridium difficile* infection [81], inflammatory bowel disease [82], and even obesity [83]. The primary mechanism of action centers on the reestablishment of a balanced microbial ecosystem [84]. The transplanted healthy microbiota can outcompete and suppress pathogenic bacteria, reestablishing microbial diversity, and restoring the integrity of the gut barrier function [85]. Additionally, the transplanted microbiota can correct aberrant metabolic pathways and restore the production of essential metabolites such as short-chain fatty acids, which have been implicated in modulating immune responses and overall gut health [86]. In this way, FMT can potentially rectify dysbiosis and associated health complications in children, although further research is required to optimize its use and understand long-term outcomes.

Dietary approaches have been suggested as an important strategy to manipulate the gut microbiota. Research has demonstrated that a diet rich in fiber can enhance the presence of many advantageous gut microbes such as *Prevotella* spp. [87]. In contrast, a Western-style diet has been associated with a decline in gut microbiota diversity and a rise in inflammation [88]. Therefore, diet interventions, such as the adoption of a Mediterranean-style diet, may be a potential approach to preventing and treating gut microbiota-related conditions [89].

Breastfeeding is another of these factors that has been shown to have a significant impact on the development of the microbiota during the early years of infant life, because it provides the infant with essential nutrients [90,91]. Several other studies reported that the most common types of intestinal flora in the breastfeeding group of infants are *Bifidobacterium* [92] and *Enterobacteriaceae* [91]. Therefore, to promote the proliferation of diversity in this microecosystem, breastfeeding could be beneficial. A cross-sectional study examined factors that influence the duration of breastfeeding in 1,008 mothers with children between 9 and 14 months of age [93]. The study shows that perinatal education of mothers increases breastfeeding duration. Conventional teaching of pregnant mothers and active pursuing of perinatal education represent key factors. Early sustained education is necessary to create favorability for breastfeeding. The study suggests that a sustained educational process before and after birth is necessary to create a general favorability for breastfeeding, which has an impact on future composition of the gut microbiota. One the other hand, formula-fed infants presented increased abundance of *Bacteroides* plus other bacteria such as *Clostridium* and *Lactobacillus* [94]. Other studies found that fecal analysis from formula-fed infants is more likely to detect *Staphylococcus*, *Escherichia coli*, and *Clostridia* [95,96]. Some interventions for the prevention and treatment of gut-microbiota-related allergies are described in Table 1.

## 7. The Relationship between Asthma, Atopic Dermatitis, and the Microbiome

**Atopic dermatitis** (AD) is a chronic skin condition characterized by recurring eczematous lesions and severe itching. The onset typically occurs in childhood, leading to its original classification as a pediatric disorder. However, recent reports have illustrated that atopic dermatitis is increasingly prevalent among adults [97,98]. The presence of bacteria in the intestine during the first three years of life can significantly affect the immune system of the host, which can have long-term effects on the host’s health and susceptibility to disease. Research has demonstrated that the development of a healthy immune system is largely reliant on the presence of intestinal bacteria, as evidenced by the weakened immune function observed in mice raised in a germ-free environment [99,100]. Thus, this condition has been associated with the development of allergic rhinitis, asthma, and food allergies [101]. In many developed countries, this disease affects over 20% of children, with an onset rate of 21.5% within the first two years of life [102,103]. In individuals with atopic dermatitis, there appears to be a correlation between a reduction in the diversity of the microbiome and the severity of the condition. An increase in colonization by harmful bacteria such as *Staphylococcus aureus* has been observed as well [104]. This is further supported by a literature review that included 44 published reports and concluded that microbiota disturbances play an important role in the proliferation of atopic dermatitis and that proper administration of probiotics could be a novel prophylactic therapy [105]. However, more research is needed to validate these results.

**Asthma** is a chronic respiratory disease that affects over 300 million individuals worldwide, making it one of the most common respiratory conditions [106]. Despite efforts to reduce the prevalence, asthma remains a significant public health concern, particularly due to high incidence in early childhood and the difficulties in management at this age [107,108,109]. Recent research has highlighted the importance of the gut microbiome in shaping the development of respiratory diseases, such as asthma, through a phenomenon known as the “gut–lung axis” [110,111,112]. It was found that the composition of the gut microbiota in children at one year of age was strongly associated with an increased risk of developing asthma by the age of five [113]. This relationship was particularly pronounced in children with a family history of asthma, suggesting that genetic predisposition and inadequate gut microbiota stimulation can work together to increase the likelihood of developing asthma. A review article highlighted that the microbiome present in the airway and/or gut could serve as a promising target for preventing or treating allergic asthma and other conditions where adaptive immune dysfunction plays a significant role [114]. Additionally, several studies have demonstrated that exposure to a wide range of microbial flora in the environment may offer protection against allergic inflammation and promote asthma [115,116]. A study conducted by Hrusch et al. [117] compared two different agricultural communities in the United States—the Amish and the Hutterites—who shared similar lifestyles but employed different farming practices. The research found that the incidence of asthma and allergic sensitization was substantially lower in the Amish, with a four- and six-times lower rate, respectively. Furthermore, the median endotoxin levels found in the dust of Amish homes were 6.8 times higher than those of Hutterite houses. In addition, immune response showed variations between the two groups. Differences in T-cell phenotypes and serum immunoglobulin E levels between Amish and Hutterite children, who live in different environments, indicate that environmental exposures can influence the development of the immune system.

## 8. The Relationship between the Microbiome and Food Allergies

Food allergy (FA) is characterized as an immune system response that is detrimental to particular dietary antigens. In recent decades, there has been an observable increase in the incidence of FA, which has become a significant health problem, affecting a notable proportion of adult and child populations, with nearly 5% of adults and 8% of children affected [118]. Cow milk and egg allergies are commonly identified as the top food allergens for young children [119,120], with egg allergy showing higher prevalence in Asia and Australia, while milk allergy is more frequently reported in the US and Middle East. While allergies to eggs, soybeans, wheat, and cow milk are typically outgrown as an individual ages, allergies to peanuts, seeds, seafood, and tree nuts tend to persist into adulthood [121]. The cause of food allergies continues to elude researchers despite extensive investigation. However, a growing body of evidence points towards the involvement of the diversity and levels in the increasing incidence of food allergies [122]. 

The research conducted by Fazlollahi et al. studied a specific food allergy in a pediatric cohort, which centered on 141 children between the ages of 3 and 16 months. The participants were sourced from five different centers located in the United States. The principal aim of the study was to scrutinize and contrast the gut microbiomes of subjects with egg allergies versus those of control subjects [123]. The findings of the investigation explained that of the 141 participating children, 66 were diagnosed with egg allergies and they exhibited an increase in the abundance of the *Lachnospiraceae* and *Streptococcaceae* families. In contrast, the control group, consisting of non-food allergic children, presented a higher occurrence of *Leuconostocaceae*. Furthermore, egg sensitization was correlated with greater gut microbiome diversity, and the *Lachnospiraceae* and *Ruminococcaceae* genera were pivotal in this regard. However, neither the differences in gut bacterial diversity nor the compositional variations in the gut microbiome could anticipate the persistence or resolution of egg allergy at 8 years of age.

In contrast, a different study focused on exploring the link between the composition of the gut microbiota in early life and the resolution of cow’s milk allergy. Research showed that infant intestinal flora in subjects between 3 and 6 months of age exhibited an increase in *Clostridia* and *Firmicutes* in those whose milk allergy improved later in childhood [124]. 

There are many potential mechanisms involved in the relationship between the microbiota and allergies; one of them is immune system modulation in which the gut microbiota can modulate the immune system by inducing regulatory T-cells (Tregs) and promoting the production of anti-inflammatory cytokines such as IL-10 [125,126]. Tregs help maintain immune tolerance to harmless antigens, such as food proteins. In individuals with a disrupted gut microbiome, the reduced production of Tregs and anti-inflammatory cytokines may lead to the loss of immune tolerance and the development of food allergies [127]. 

Another potential mechanism is the function of the gut barrier; the gut microbiome contributes to the maintenance of the gut barrier function, which prevents the entry of harmful substances and pathogens into the bloodstream [128]. Dysbiosis can potentially lead to a compromised gut barrier, allowing food allergens to cross the gut epithelium and provoke an immune response. Necrotizing enterocolitis (NEC) is a common disease among preterm infants and is considered the most common and severe emergency in neonatal intensive care units [129]. It is characterized by widespread intestinal tissue necrosis together with increased abundance of proinflammatory cytokines. Bacteremia and endotoxemia have also been common [130]. NEC is a condition that significantly contributes to illness and death among premature newborns [131,132]. Multiple risk factors have been identified in the development of this disease, one of particular interest being formula feeding, specifically high-osmotic-strength formula feeding, which has been identified as a primary risk factor [133]. The gut microbiota of infants who develop NEC exhibits significant differences [134,135]. Previous culture-based studies reported changes in fecal bacteria as early as 72 h before the onset of NEC [136]. The observed alterations in fecal bacteria included elevated levels of *Enterobacter cloacae* and *Escherichia coli*, alongside decreased levels of *Streptococcus faecalis* and *Staphylococcal* species. Due to increased permeability in the intestines of preterm infants, it can be considered that this increase will lead to increased translocation of bacteria and explain bacteremia and endotoxemia in these premature infants. A study that observed the gut microbiota of preterm twins over time revealed a decline in the diversity of the gut microbiota and a rising prevalence of *Escherichia* spp. prior to the onset of NEC. Conversely, this pattern was not observed in healthy twins who did not develop NEC later [137]. Another follow-up study discovered notable variations in the microbiota composition of individuals with NEC compared to controls, one week before the NEC diagnosis [138]. Within the NEC cases, there was a 34% increase in the *Proteobacteria* population and a 32% decrease in the population of *Firmicutes* between the samples collected one week earlier and less than 72 h before the onset of NEC. In addition, not only preterm birth increases the risk factor, but studies have suggested that even antibiotic use and formula feeding have been associated with the development of NEC [139,140]. The relationship between the human microbiome and food allergies in children is complex and multifactorial. The gut microbiome plays a crucial role in the formation of the immune system, and alterations in the microbial community can increase the risk of developing food allergies. Understanding the mechanisms involved in this relationship can help in the development of novel therapeutic strategies to prevent and treat food allergies in children.

## 9. Conclusions

In conclusion, this literature review has highlighted the vital role that the gut microbiota plays in the development and manifestation of allergies in children. The complexity of the human gut microbiota, which is influenced by factors such as host genotype, mode of delivery, breastfeeding, diet, antibiotic use, and environmental factors, underscores the intricacies involved in establishing a healthy microbial ecosystem. The association between the gut microbiota and atopic symptoms or sensitization, immune system development, body growth, and various diseases such as atopic dermatitis, asthma, and necrotizing enterocolitis demonstrates the far-reaching implications of a balanced gut microbiome. Interventions such as probiotics, prebiotics, fecal microbiota transplantation, dietary approaches, and breastfeeding have shown promising results in preventing and treating gut-microbiota-related allergies. 

Notably, the adoption of a Mediterranean-style diet, probiotics, and prebiotics may help to mitigate the risk of developing allergies, while breastfeeding is essential in establishing a diverse gut microbiome and providing critical nutrients to infants. Furthermore, the relationship between the gut microbiota and food allergies highlights the potential to develop novel treatments that target the gut microbiota to improve intestinal barrier function and modulate the immune response to food allergens. Despite the advances in our understanding of the gut microbiota and its implications on allergies in children, further research is warranted to elucidate the complex interplay between the microbiome, immune system, and disease development. By deepening our knowledge in this area, we can pave the way for more targeted and effective interventions that could potentially transform the prevention and management of allergies in children.

## Figures and Tables

**Table 1:** Overview of Different Interventions and Their Role in the Gut Microbiota.

Intervention	Primary Function	Examples of Health Benefits	Studies
Probiotics	Restoration of microbial equilibrium	Prevents and treats gut-related disorders (e.g., antibiotic-associated diarrhea, IBD, celiac disease), reduces symptoms of allergic rhinitis	[62,63,64,65,66,67,68,69,70,71,72,73]
Prebiotics	Promotes growth of beneficial gut bacteria	Improves gut barrier function, enhances immune response, regulates host metabolism, reduces risk of allergies	[74,75,76,77,78]
Fecal microbiota transplantation	Replaces gut microbiota with healthy microbiota	Effective in treating recurrent Clostridium difficile infection, IBD, obesity	[79,80,81,82,83,84,85]
Dietary approaches	Manipulates gut microbiota	Enhances beneficial gut microbes, reduces inflammation	[86,87,88]
Breastfeeding	Provides essential nutrients for infant microbiota development	Promotes proliferation of gut microecosystem, increases breastfeeding duration	[89,90,91,92,93,94,95]
